# Weight loss maintenance after tirzepatide cessation in people with overweight/obesity: a real-world follow-up of the phase 3 SURMOUNT-CN trial

**DOI:** 10.1093/lifemeta/loaf024

**Published:** 2025-06-12

**Authors:** Congling Chen, Zhen Ying, Qi Tang, Lin Zhao, Yibing Lu, Xiaofang Fan, Lan Xu, Si Si, Yuanyuan Li, Jiawei Xu, Yihua Wang, Yu Dong, Xiaoying Li, Ying Chen

**Affiliations:** Department of Endocrinology and Metabolism, Zhongshan Hospital, Fudan University, Shanghai 200032, China; Department of Endocrinology and Metabolism, Zhongshan Hospital, Fudan University, Shanghai 200032, China; Department of Endocrinology and Metabolism, Zhongshan Hospital, Fudan University, Shanghai 200032, China; Department of Endocrinology and Metabolism, Zhongshan Hospital, Fudan University, Shanghai 200032, China; Department of Endocrinology, The Second Affiliated Hospital of Nanjing Medical University, Nanjing, Jiangsu 210011, China; Department of Endocrinology, Minhang Hospital, Fudan University, Shanghai 201199, China; Department of Endocrinology, Wuxi People’s Hospital, Nanjing Medical University, Wuxi, Jiangsu 214023, China; Eli Lilly and Company, Shanghai 200041, China; Eli Lilly and Company, Shanghai 200041, China; Eli Lilly and Company, Shanghai 200041, China; Eli Lilly and Company, Shanghai 200041, China; Eli Lilly and Company, Shanghai 200041, China; Department of Endocrinology and Metabolism, Zhongshan Hospital, Fudan University, Shanghai 200032, China; Department of Endocrinology and Metabolism, Zhongshan Hospital, Fudan University, Shanghai 200032, China

**Keywords:** obesity, overweight, tirzepatide, GLP-1, GIP, body weight

## Abstract

Tirzepatide, a dual GIP/GLP-1 receptor agonist, has shown unprecedented efficacy in weight loss and improving metabolic parameters in clinical trials. However, the durability of these benefits after treatment cessation remains poorly understood. This study aimed to investigate the effect of tirzepatide cessation on body weight change in people with obesity or overweight. This real-world, observational 26-week follow-up study included participants who completed 52 weeks’ study treatment during SURMOUNT-CN (NCT05024032). The analysis excluded participants who received anti-obesity medications or bariatric procedures during the 26-week follow-up. Key outcomes were the changes in body weight and waist circumference after 26 weeks, from treatment cessation (Week 52) and trial baseline (Week 0), by the SURMOUNT-CN treatment groups (tirzepatide 10 or 15 mg, or placebo). Overall, 152 participants were included (tirzepatide 10 mg: *n* = 57; tirzepatide 15 mg: *n *= 51; placebo: *n *= 44). Following treatment cessation (Week 52), the mean (SD) percentage changes in body weight at Week 78 were 9.1% (7.4), 12.3% (9.9), and 1.8% (5.2), and the absolute changes in waist circumference were 2.9 cm (7.5), 5.5 cm (6.5) and −0.5 cm (5.6), in tirzepatide 10 and 15 mg, and placebo groups, respectively. From trial baseline to Week 78, the mean (SD) net percentage weight changes were −8.7% (6.9), −10.6% (10.2), and −2.5% (7.0), and the changes in waist circumference were −10.5 cm (8.1), −10.6 cm (9.3) and −4.0 cm (7.2), in the tirzepatide 10 and 15 mg, and placebo groups, respectively. At Week 78, residual improvements in multiple cardiometabolic indicators were evident in the tirzepatide groups. Despite weight gain following tirzepatide cessation, participants achieved a large net weight loss and reduction in waist circumference from trial baseline to Week 78, with residual improvements in several cardiometabolic indicators.

## Introduction

Against the backdrop of a rapidly progressing global obesity epidemic, emerging evidence reveals an interplay between obesity, obesity-driven inflammation, and downstream complications [1]. Obesity is a chronic, progressive, and recurrent disease caused by a combination of genetic and environmental factors, characterized by an excessive accumulation or abnormal distribution of fat tissue, leading to dysfunction [1–4].

Lifestyle interventions are the first-line approach for achieving weight loss [5, 6]. However, people with obesity or overweight receiving these approaches usually fail to maintain long-term weight loss [7]. It is well documented that the cessation of weight management strategies, such as discontinuation of behavioral support and termination of anti-obesity medications (AOMs), leads to weight regain [3, 4]. Weight regain is not only driven by poor adherence to lifestyle interventions during the extended periods but also by the nature of adaptive mechanisms to the weight loss by reducing rest energy expenditure [3, 4]. Therefore, obesity has been recognized as a chronic relapsing disease that requires lifelong management [8]. International and Chinese clinical guidelines recommend adjunctive pharmacotherapy as a component of clinical strategies for achieving and maintaining weight loss in individuals with overweight or obesity [6, 9–12]. Weight regain has also been observed following bariatric surgery [13]. Therefore, long-term integrated weight management is required to maintain weight loss and prevent weight regain [14–20].

While a modest reduction in body weight has been demonstrated with early-generation AOMs (5%–8%) [21–23], evidence suggests that greater weight loss is required for some patients with obesity-related comorbid conditions, such as obstructive sleep apnea and metabolic dysfunction-associated steatohepatitis, highlighting the urgency for AOMs with a stronger weight loss effect (> 15% weight loss) [24]. Tirzepatide is a once-weekly dual glucose-dependent insulinotropic polypeptide and glucagon-like peptide-1 (GIP/GLP-1) receptor agonist that exerts synergistic effects on food intake, appetite, and metabolic function [25–27]. In international clinical trials, treatment with tirzepatide 10 or 15 mg led to significant and sustained reductions in body weight of up to 22.5% over 72 weeks of treatment [18, 28, 29]. The SURMOUNT-4 trial demonstrated that tirzepatide 10 or 15 mg plus lifestyle intervention for 36 weeks resulted in a mean weight reduction of 20.9% and an additional 5.5% weight reduction from Week 36 to 88, while participants who switched to placebo experienced a 14.0% weight regain. Continuous tirzepatide treatment was also associated with metabolic benefits, including significant improvements from Week 36 to 88 in body mass index (BMI), hemoglobin A1c (HbA1c), fasting glucose, insulin, lipid levels, and systolic and diastolic blood pressure, compared with placebo [18].

The phase 3 SURMOUNT-CN trial demonstrated the efficacy and safety of tirzepatide 10 or 15 mg for weight reduction in Chinese adults with obesity or overweight over 52 weeks of treatment [30]. In this trial, while the definitions of overweight and obesity (BMI 24−28 kg/m^2^ and ≥ 28 kg/m^2^, respectively) [30] were lower than those used in other global phase 3 trials of tirzepatide (BMI ≥ 27 kg/m^2^ and ≥ 30 kg/m^2^, respectively) [18, 28, 29], these thresholds are used in clinical practice in China [2]. In addition, to date, weight loss maintenance following tirzepatide withdrawal has not been investigated in real-world settings. Therefore, in this follow-up extension of SURMOUNT-CN, we investigated changes in body weight, waist circumference, and other cardiometabolic parameters for 6 months following tirzepatide cessation.

## Results

### Participants

A total of 160 participants completed the 52-week SURMOUNT-CN trial from April 2023 to July 2023 and consented to be included in this follow-up study, among whom 152 formed the eligible analysis population (EAP) (tirzepatide 10 mg: *n *= 57; tirzepatide 15 mg: *n *= 51; placebo: *n* = 44) (Supplementary Fig. S1). All 152 participants completed the follow-up study.

At trial baseline (Week 0), the 152 participants included in this analysis had a mean (standard deviation (SD)) age of 36.0 years (9.1) and 46.7% were female, with a mean (SD) body weight of 92.5 kg (16.0), a BMI of 32.4 kg/m^2^ (3.8), and a waist circumference of 104.8 cm (10.2) (Supplementary Table S1). Overall, 67% of waist circumference measurements were made in the clinic. At trial baseline, over 90% of participants in all treatment groups had obesity (BMI ≥ 28 kg/m^2^), and the two most prevalent comorbidities were dyslipidemia (75.7%) and hypertension (25.7%).

The treatment outcomes from trial baseline to Week 52 in this analysis population were consistent with the findings in the primary analysis population (*n* = 210, presented in the primary publication [30]). Briefly, greater mean (SD) percentage changes in body weight from trial baseline to Week 52 were observed for participants in the tirzepatide 10 and 15 mg groups versus placebo group: −15.3% (8.0), −19.9% (8.8), and −3.8% (6.1), respectively (Supplementary Table S2). More than half of the participants who received tirzepatide 10 or 15 mg (54.4% and 68.6%, respectively) achieved ≥ 15% weight loss from baseline at Week 52 (Supplementary Table S2). Similarly, greater mean changes in waist circumference from trial baseline to Week 52 were observed for participants in the tirzepatide 10 and 15 mg groups versus the placebo group (Supplementary Table S3).

## Changes in body weight and waist circumference in the real-world follow-up

**Figure 1:**
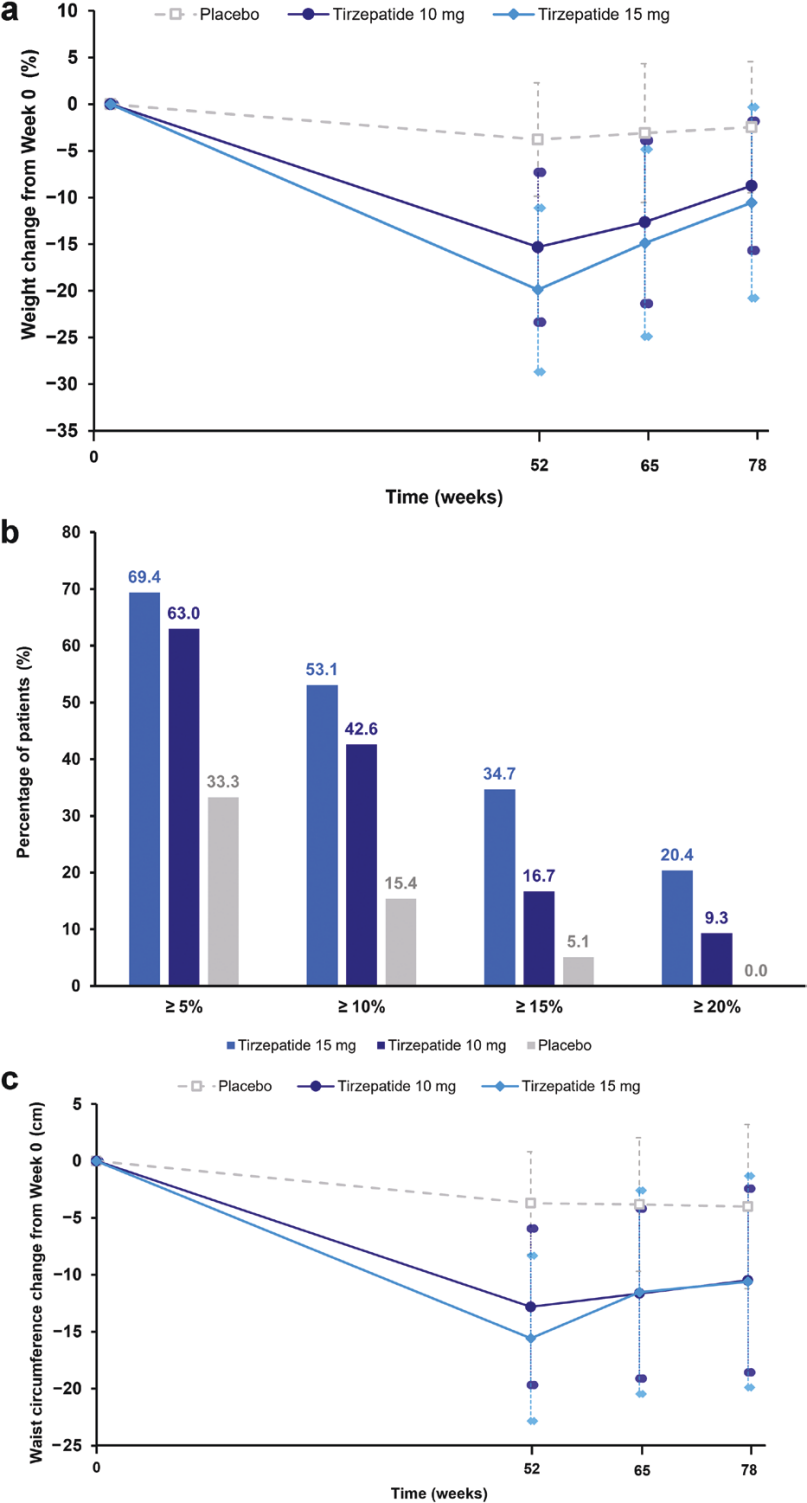
(a) Mean (SD) values of percentage change in body weight from trial baseline (Week 0) to Weeks 52, 65, and 78. (b) The proportion of participants achieving weight loss categories from trial baseline to Week 78. (c) Mean (SD) values of absolute change in waist circumference from trial baseline to Weeks 52, 65, and 78. SD, standard deviation.

Among participants in the EAP, from Week 52 to 78, the mean (SD) percentage changes in body weight in the tirzepatide 10 and 15 mg, and placebo groups were 9.1% (7.4), 12.3% (9.9), and 1.8% (5.2), respectively (Supplementary Table S4). From trial baseline to Week 78 (26-week post-trial follow-up), the mean (SD) percentage changes in body weight were −8.7% (6.9), −10.6% (10.2), and −2.5% (7.0), respectively (Fig. 1a; Supplementary Table S2), and the corresponding mean (SD) absolute changes in body weight were −8.1 kg (6.7), −9.7 kg (9.8), and −2.1 kg (6.6), in the tirzepatide 10 and 15 mg, and placebo groups, respectively. At Week 78, a higher proportion of participants receiving tirzepatide 15 and 10 mg had achieved ≥ 10% (53.1% and 42.6% versus 15.4%) and ≥ 15% (34.7% and 16.7% versus 5.1%) weight loss from trial baseline versus placebo (Fig. 1b).

The mean (SD) absolute changes in waist circumference from Week 52 to 78 were 2.9 cm (7.5), 5.5 cm (6.5), and −0.5 cm (5.6) in the tirzepatide 10 and 15 mg, and placebo groups, respectively (Supplementary Table S5). From trial baseline to Week 78, participants in the tirzepatide 10 and 15 mg groups achieved mean reductions in waist circumference of −10.5 cm (8.1) and −10.6 cm (9.3), respectively, compared with −4.0 cm (7.2) in the placebo group (Fig. 1c).

### Cardiometabolic risk factors

Significant improvements in all cardiometabolic risk factors evaluated were observed at Week 52 (Table 1). Following tirzepatide cessation, residual improvements were observed at Week 78. At Week 78, significantly different changes from trial baseline favoring the tirzepatide treatment groups were observed for total cholesterol (*P* = 0.04), and low-density lipoprotein (LDL) (*P* = 0.03). Borderline significance was observed for the change in HbA1c in participants receiving tirzepatide versus placebo in a three-group analysis (*P* = 0.06). When the two tirzepatide treatment groups were combined, significant differences were evident between the tirzepatide-treated groups and the placebo group (*P* = 0.02).

**Table 1.** Changes in cardiometabolic factors from trial baseline (EAP).

	Tirzepatide 10 mg		Tirzepatide 15 mg		Placebo		*P*-value
SBP < 120 and DBP < 80 mmHg	*n*	*n* (%)	*n*	*n* (%)	*n*	*n* (%)	
Week 52	56	35 (62.5)	49	34 (69.4)	42	17 (40.5)	0.02
Week 78	49	18 (36.7)	48	20 (41.7)	38	11 (28.9)	0.47
**Change from baseline**	***n***	**Mean (SD)**	***n***	**Mean (SD)**	***n***	**Mean (SD)**	
HbA1c (%)							
Week 52	56	−0.4 (0.3)	49	−0.3 (0.3)	42	0.0 (0.3)	< 0.01
Week 78	17	−0.3 (0.3)	19	−0.3 (0.2)	16	−0.1 (0.3)	0.06
Total cholesterol (mmol/L)							
Week 52	56	−0.3 (0.6)	49	−0.3 (0.6)	42	−0.1 (0.9)	0.33
Week 78	21	0.1 (0.6)	23	−0.4 (1.1)	21	0.2 (0.7)	0.04
Triglycerides (mmol/L)							
Week 52	56	−0.8 (0.9)	49	−0.6 (1.0)	42	0.5 (4.9)	0.06
Week 78	22	−0.3 (0.6)	23	−0.1 (1.0)	21	0.1 (1.1)	0.30
HDL (mmol/L)							
Week 52	56	0.1 (0.2)	49	0.1 (0.2)	42	0.0 (0.2)	0.01
Week 78	22	0.2 (0.2)	23	0.2 (0.2)	21	0.1 (0.2)	0.15
LDL (mmol/L)							
Week 52	56	−0.1 (0.6)	49	−0.2 (0.5)	42	−0.2 (0.7)	0.83
Week 78	22	0.0 (0.6)	23	−0.4 (0.9)	21	0.3 (0.6)	0.03

DBP, diastolic blood pressure; EAP, eligible analysis population; HbA1c, hemoglobin A1c; HDL, high-density lipoprotein; LDL, low-density lipoprotein; SBP, systolic blood pressure; SD, standard deviation.

### Compliance with lifestyle interventions

The majority of responding participants reported either partial or poor compliance with lifestyle consultations during routine care from Week 52 to 78 (Table 2). Only 7.2% of all participants overall reported complete compliance. The rates of complete or partial adherence to lifestyle consultations were numerically higher among participants in the tirzepatide 10 and 15 mg groups compared with the placebo group at Week 78 (40.4% and 49.0% versus 34.1%, respectively).

**Table 2.** Compliance to lifestyle consultations at Week 78.

*n* (%)	Tirzepatide 10 mg (*n *= 57)	Tirzepatide 15 mg (*n *= 51)	Placebo (*n *= 44)	Total (*n *= 152)
Complete compliance	4 (7.0)	4 (7.8)	3 (6.8)	11 (7.2)
Partial compliance	19 (33.3)	21 (41.2)	12 (27.3)	52 (34.2)
Poor compliance	14 (24.6)	12 (23.5)	7 (15.9)	33 (21.7)
No compliance	1 (1.8)	1 (2.0)	3 (6.8)	5 (3.3)
No lifestyle consultation	18 (31.6)	13 (25.5)	17 (38.6)	48 (31.6)
Missing data	1 (1.8)	0	2 (4.5)	3 (2.0)

## Regression analysis

In the multivariate regression analysis, percentage weight loss during the treatment period (the first 52 weeks) and percentage weight regain at Week 56 from Week 52 were positively associated with the magnitude of weight change from Week 52 to Week 78 (Table 3). Patient-reported partial to complete compliance to lifestyle consultations was negatively associated with weight change. No association was observed between weight change from Week 52 to 78 and the other factors investigated, including patient sex, age, baseline BMI, hypertension, dyslipidemia, obstructive sleep apnea, and atherosclerotic cardiovascular disease.

**Table 3.** Multivariate linear regression analysis of percentage weight change from Week 52 to Week 78.

	Multivariate regression		
	Coefficient	SE	*P*-value
**Sex**			
Female	1.00		
Male	0.45	1.40	0.75
**Age (continuous)**	0.06	0.08	0.44
**Baseline BMI (continuous)**	−0.22	0.18	0.23
**Hypertension at baseline**	1.64	1.59	0.30
**Dyslipidemia at baseline**	−1.42	1.56	0.36
**OSA at baseline**	−4.70	2.92	0.11
**ASCVD at baseline**	1.47	4.24	0.73
**Weight loss group at Week 52**			
< 5%	1.00		
5%−10%	3.01	2.08	0.15
10%−15%	4.89	2.10	0.02
15%−20%	7.82	2.06	< 0.01
> 20%	11.93	2.08	< 0.01
**Weight regain at Week 56 (% change from Week 52)**	1.03	0.27	< 0.01
**Lifestyle consultation**			
No consultation/no compliance	1.00		
Poor compliance	−1.43	1.73	0.41
Partial compliance	−3.12	1.55	0.04
Complete compliance	−6.56	2.54	0.01

ASCVD, atherosclerotic cardiovascular disease; BMI, body mass index; OSA, obstructive sleep apnea; SE, standard error.

## Discussion

In the SURMOUNT-CN trial, Chinese participants with obesity or overweight receiving tirzepatide 10 or 15 mg for 52 weeks achieved significantly higher reductions in body weight (up to 19.9%) and waist circumference (up to −16.4 cm) versus those receiving placebo [30]. In this observational follow-up study, the net percentage changes in body weight from trial baseline to Week 78 (26-week post-trial follow-up) among participants who received tirzepatide 10 or 15 mg were −8.7% and −10.6%, respectively. Furthermore, a higher proportion of participants who received tirzepatide 10 or 15 mg achieved a net weight loss from trial baseline to Week 78 of ≥ 10% (42.6% and 53.1% versus 15.4%, respectively) or ≥ 15% (16.7% and 34.7% versus 5.1%, respectively), compared with placebo. This is the first investigation of weight loss maintenance after tirzepatide cessation in Chinese people with overweight or obesity defined using BMI cutoffs of 24−28 kg/m^2^ and ≥ 28 kg/m^2^. Given that these BMI cutoffs are commonly utilized in clinical practice in China [2], our results will inform the clinical decision-making and management of people with obesity or overweight in China. Furthermore, these China-specific BMI cutoffs reflect the higher risk of mortality and cardiovascular disease observed in Chinese populations versus Western populations at the same BMI [2]. Using these BMI cutoffs may therefore increase comparability with the results of international trials such as the SURMOUNT-4 trial [18].

Compared with the STEP 1 extension study (a follow-up study of a phase 3 trial of weekly semaglutide 2.4 mg for weight management) [16], the present study demonstrated a comparable trend of weight change, with higher percentage change from baseline in body weight in both tirzepatide groups compared with the placebo group by the end of the study (−8.7% and −10.6% versus −2.5%). This may relate to the relatively short follow-up period of the present study. More importantly, the results of the present study are consistent with the findings from the phase 3 SURMOUNT-4 trial, in which predominantly White participants with obesity or overweight received tirzepatide 10 or 15 mg for 36 weeks before being randomized to either continue tirzepatide at the same dose or receive placebo for a further 52 weeks [18]. Participants in the SURMOUNT-4 trial who switched from tirzepatide 10 or 15 mg to placebo for 52 weeks experienced an increase in body weight after discontinuing tirzepatide but achieved a net 9.9% reduction in mean body weight from trial baseline, with 46.2% achieving a ≥ 10% weight reduction. Although the SURMOUNT-4 trial utilized different treatment durations and a different length of open-label extension compared to the present study, the results were broadly comparable to the net mean weight reduction (−8.7% to −10.6%) and proportion of participants achieving body weight reductions of ≥ 10% (42.6% to 53.1%) with tirzepatide observed in the present study from trial baseline at Week 78. The results of the SURMOUNT-4 trial also showed that, after 36 weeks’ treatment with tirzepatide 10 or 15 mg, extending tirzepatide treatment for 52 weeks resulted in a further 5.5% weight loss, 4.3 cm reduction in waist circumference, and further improvements in cardiometabolic factors, including BMI, HbA1c, fasting glucose, lipid profile, and blood pressure [18].

In addition to a net mean body weight loss, participants in the present study receiving tirzepatide 10 or 15 mg also achieved a net reduction in mean waist circumference from trial baseline to Week 78 (−10.5 and −10.6 cm, respectively). Consistent with this finding, participants in the SURMOUNT-4 trial who received tirzepatide 10 or 15 mg for 36 weeks before being randomized to placebo for a further 52 weeks also achieved net reductions in mean body weight (−9.5%) and waist circumference (−9.1 cm) by Week 88 [18]. These findings suggest that the reduction of adipose tissue achieved during treatment with tirzepatide is partially maintained following treatment cessation for at least 26 weeks, but changes over the longer-term require further investigation. This is highly clinically relevant in China as it is estimated that 26.0% of adults with a normal BMI have abdominal obesity (waist circumference ≥ 90 cm in men and ≥ 80 cm in women) [31]. In addition, waist circumference is an independent risk factor for multiple comorbidities, such as type 2 diabetes and cardiovascular disease [32, 33].

There is generally a positive association between the magnitude of weight loss and reduction in comorbidity risk in individuals with overweight or obesity [10, 24]. In the present study, reductions in cardiometabolic risk factors achieved during tirzepatide treatment were partially maintained following tirzepatide cessation (from trial baseline to Week 78), including net reductions in mean HbA1c, total cholesterol, and LDL cholesterol. Our results are generally consistent with those from the SURMOUNT-4 trial among participants who switched to placebo for 52 weeks after receiving 36 weeks of tirzepatide [18]. Long-term maintenance of weight loss is necessary and is associated with a lower incidence and delayed onset of cardiometabolic diseases in individuals with overweight or obesity [34].

Given that obesity has a complex etiology, long-term weight loss success is infrequently achieved with a diet- or exercise-only intervention [35]. The results of our regression analysis suggest that partial and complete compliance with lifestyle consultations during routine care may reduce or slow weight increase after tirzepatide cessation. However, although up to 70% of all participants received lifestyle consultations, only 7% reported complete compliance. This emphasizes the practical limitations of using lifestyle interventions to maintain body weight loss after a pharmacological weight loss intervention. Therefore, long-term integrated weight management (adjunctive pharmacotherapy combined with lifestyle modifications) is essential in clinical practice to ensure a greater magnitude and longer maintenance of weight loss and a corresponding sustained reduction in cardiometabolic risk factors [35]. In addition, our study results suggest that the magnitude of weight increase after tirzepatide cessation (Week 52 to 78) is significantly associated with the immediate weight increase following cessation (Week 52 to 56). These results emphasize the importance of close monitoring and continuous weight management following adjustments of weight management plans and AOM treatment. In summary, given the chronic nature of obesity, persistent integrated weight management with combined AOM usage and lifestyle intervention is necessary.

## Conclusions

Despite weight regain after treatment cessation, study participants exhibited a net weight loss from trial baseline to Week 78, 26 weeks after tirzepatide cessation. Compliance to lifestyle consultations was associated with less weight regain. However, the real-world compliance rate was low in this study. Finally, at Week 78, residual improvements in several cardiometabolic indicators were observed in participants who had received tirzepatide.

## Limitations of the study

This study has several limitations. First, due to interruptions during the COVID-19 pandemic, there was a high level of missing data at the Week 65 visit. Second, the 26-week follow-up was relatively short, and further studies are required to evaluate the long-term effects after tirzepatide cessation. Third, this follow-up extension included participants who completed the SURMOUNT-CN trial, agreed to participate, and did not receive AOM during the post-trial period. This limited the sample size and only permitted descriptive evaluation of exploratory endpoints. However, the results of the present study are generally consistent with the findings of the SURMOUNT-4 trial 52 weeks following tirzepatide cessation [18].

## Methods

### Study design and participants

This was a real-world, observational follow-up of the phase 3, randomized, double-blind SURMOUNT-CN trial (NCT05024032). The full methods of SURMOUNT-CN have been published elsewhere [30]. Briefly, Chinese adults (*n* = 210) with a BMI of ≥ 28 kg/m^2^, or ≥ 24 kg/m^2^ with ≥ 1 weight-related comorbidity (excluding diabetes), were randomized (1:1:1) to receive once-weekly tirzepatide 10 or 15 mg, or placebo, plus lifestyle intervention, for 52 weeks. Randomization was stratified by sex (female, male) and the presence of weight-related comorbidities (yes, no). Sex was self-reported by participants based on a choice of “male” and “female”. The primary endpoints were mean percentage change from baseline in body weight and the percentage of participants with weight reduction of at least 5% at Week 52. This 26-week follow-up study included all participants who completed 52 weeks of treatment during the SURMOUNT-CN trail and consented to participate in the follow-up study. This further analysis excluded participants who received AOM or bariatric procedures for the purpose of weight loss during the 26-week follow-up period, comprising the EAP.

The protocol for this extension phase was approved by the Ethics Review Board at Zhongshan Hospital, Fudan University. The study was conducted in accordance with the ethical principles outlined in the Declaration of Helsinki and following Good Pharmacoepidemiology Practices and applicable local laws and regulations. All participants provided written informed consent before inclusion.

### Treatment

As this was a real-world observational study, clinical management was at the discretion of managing physicians, and no intervention was imposed.

### Outcomes and measurements

The study outcomes included absolute and percentage changes in body weight and absolute waist circumference at Week 78 from Week 52 and Week 0, respectively, by SURMOUNT-CN treatment groups (tirzepatide 10 or 15 mg, or placebo), and changes in cardiometabolic indicators of interest, including blood pressure and laboratory results of fasting glucose, HbA1c, total cholesterol, triglycerides, high-density lipoprotein (HDL) cholesterol, and LDL cholesterol at Week 78.

Patient-level data at the trial baseline and Week 52 were extracted from the SURMOUNT-CN trial. Follow-up data, including body weight, waist circumference, blood pressure, and copies of laboratory reports (if available), were collected via patient self-report. Participants were strongly encouraged to have their waist circumference measured in the clinic where possible and were educated on the proper measurement of waist circumference and body weight.

### Statistical analysis

Continuous variables were summarized using mean and SD or median and range (minimum, maximum). Categorical variables were summarized using frequency and proportion. The chi-square test and analysis of variance (ANOVA) were applied to compare categorical and continuous variables between groups. Analyses were conducted using complete data only.

A multivariate linear regression analysis of percentage weight change from Week 52 to Week 78 was performed, with covariates, including patient sex (male), age, baseline BMI, hypertension, dyslipidemia, obstructive sleep apnea, atherosclerotic cardiovascular disease, weight loss from trial baseline to Week 52 (by percentage weight loss category), weight change at Week 56 (percentage change from Week 52), and self-reported complicate to lifestyle consultations. Participants with missing values in any variable were excluded.

## Supplementary Material

loaf024_suppl_Supplementary_Materials

## Data and resource availability

Subject data are recorded on data forms. Investigators are responsible for the integrity of the data (i.e., accuracy, completeness, legibility, and timeliness) reported to Lilly. The investigator follows local laws and regulations or institutional practices for document retention. All information about this observational study and individual subject medical information resulting from this study are considered confidential, and disclosure to third parties is prohibited except for regulatory authorities and as applicable by law. Publications may result from this study.
