# Supplementary material for: Weight loss maintenance after tirzepatide cessation in people with overweight/obesity: a real-world follow-up of the phase 3 SURMOUNT-CN trial
Source: Life Metab. 2025 Jun 12;4(5):loaf024. doi: 10.1093/lifemeta/loaf024 (PMC13122358; doi:10.1093/lifemeta/loaf024)
Supplement: loaf024_suppl_Supplementary_Materials [file loaf024_suppl_supplementary_materials.docx]

**SUPPLEMENTARY MATERIALS for**

**Weight loss maintenance after tirzepatide cessation in people with overweight/obesity:**

**a real-world follow-up of the phase 3 SURMOUNT-CN trial**

**Supplementary Table S1** Patient characteristics at trial baseline (Week 0; EAP).

|  | **Tirzepatide 10 mg (*n* = 57)** | **Tirzepatide 15 mg (*n* = 51)** | **Placebo (*n* = 44)** | **Total (*n* = 152)** |
| --- | --- | --- | --- | --- |
| Age (years) | 35.2 (7.5) | 36.8 (9.6) | 36.1 (10.6) | 36.0 (9.1) |
| Female sex, *n* (%) | 30 (52.6) | 24 (47.1) | 17 (38.6) | 71 (46.7) |
| Region, *n* (%) | | | | |
| Northwest China | 1 (1.8) | 2 (3.9) | 1 (2.3) | 4 (2.6) |
| Northern China | 15 (26.3) | 18 (35.3) | 14 (31.8) | 47 (30.9) |
| Southern China | 41 (71.9) | 31 (60.8) | 29 (65.9) | 101 (66.4) |
| Body weight (kg) | 92.1 (16.3) | 91.3 (15.3) | 94.5 (16.7) | 92.5 (16.0) |
| BMI (kg/m^2^) | 32.7 (4.1) | 32.0 (3.6) | 32.6 (3.6) | 32.4 (3.8) |
| BMI category at trial baseline, *n* (%) | | | | |
| ≥ 24.0 and < 28.0 kg/m^2^ | 3 (5.3) | 4 (7.8) | 3 (6.8) | 10 (6.6) |
| ≥ 28.0 and < 30.0 kg/m^2^ | 12 (21.1) | 12 (23.5) | 7 (15.9) | 31 (20.4) |
| ≥ 30.0 and < 35.0 kg/m^2^ | 26 (45.6) | 25 (49.0) | 24 (54.5) | 75 (49.3) |
| ≥ 35.0 kg/m^2^ | 16 (28.1) | 10 (19.6) | 10 (22.7) | 36 (23.7) |
| Waist circumference, cm | 104.4 (9.8) | 104.1 (10.8) | 106.0 (10.2) | 104.8 (10.2) |
| Comorbidity, *n* (%) | | | | |
| Hypertension | 15 (26.3) | 12 (23.5) | 12 (27.3) | 39 (25.7) |
| Dyslipidemia | 39 (68.4) | 39 (76.5) | 37 (84.1) | 115 (75.7) |
| Obstructive sleep apnea | 5 (8.8) | 2 (3.9) | 1 (2.3) | 8 (5.3) |
| Atherosclerotic cardiovascular disease | 4 (7.0) | 1 (2.0) | 0 | 5 (3.3) |
| Pre-diabetes | NA | NA | NA | NA |
| None of the above | 13 (22.8) | 8 (15.7) | 6 (13.6) | 27 (17.8) |

Data are mean (SD) unless otherwise specified. BMI, body mass index; EAP, eligible analysis population; NA, not applicable; SD, standard deviation.

**Supplementary Table S2** Percentage and absolute change in body weight (kg) from Week 0 (EAP).

|  | **Tirzepatide 10 mg (*n* = 57)** | **Tirzepatide 15 mg (*n* = 51)** | **Placebo (*n* = 44)** |
| --- | --- | --- | --- |
| **Change from Week 0 to Week 52** | | | |
| *n* | 56 | 49 | 42 |
| Percentage change (%) | −15.326 (8.038) | −19.884 (8.776) | −3.798 (6.083) |
| Absolute change (kg) | −13.893 (7.176) | −18.078 (8.361) | −3.464 (5.706) |
| **Change from Week 0 to Week 56** | | | |
| *n* | 54 | 48 | 40 |
| Percentage change (%) | −12.815 (7.187) | −17.869 (8.882) | −2.871 (6.576) |
| Absolute change (kg) | −11.680 (6.491) | −16.233 (8.436) | −2.615 (6.262) |
| **Change from Week 0 to Week 78** | | | |
| *n* | 54 | 49 | 39 |
| Percentage change (%) | −8.738 (6.927) | −10.567 (10.231) | −2.472 (7.032) |
| Absolute change (kg) | −8.012 (6.689) | −9.688 (9.825) | −2.119 (6.647) |
| **Weight loss category from Week 0 to Week 52** | | | |
| < 5% | 4 (7.0) | 3 (5.9) | 24 (54.5) |
| ≥ 5% and < 10% | 11 (19.3) | 4 (7.8) | 10 (22.7) |
| ≥ 10% and < 15% | 10 (17.5) | 7 (13.7) | 6 (13.6) |
| ≥ 15% and < 20% | 16 (28.1) | 12 (23.5) | 2 (4.5) |
| ≥ 20% | 15 (26.3) | 23 (45.1) | 0 |
| **Weight loss category from Week 0 to Week 78** | | | |
| *n* | 54 | 49 | 39 |
| < 5% | 20 (37.0) | 15 (30.6) | 26 (66.7) |
| ≥ 5% and < 10% | 11 (20.4) | 8 (16.3) | 7 (17.9) |
| ≥ 10% and < 15% | 14 (25.9) | 9 (18.4) | 4 (10.3) |
| ≥ 15% and < 20% | 4 (7.4) | 7 (14.3) | 2 (5.1) |
| ≥ 20% | 5 (9.3) | 10 (20.4) | 0 |

Data are mean (SD) unless otherwise specified. EAP, eligible analysis population; SD, standard deviation.

Supplementary Table S3 Absolute change in waist circumference (cm) from Week 0 (EAP)

|  | **Tirzepatide 10 mg (*n* = 57)** | **Tirzepatide 15 mg (*n* = 51)** | **Placebo (*n* = 44)** |
| --- | --- | --- | --- |
| **Change from Week 0 to Week 52** | | | |
| *n* | 56 | 49 | 42 |
| Absolute change (cm) | −12.817 (6.863) | −15.592 (7.257) | −3.702 (4.513) |
| **Change from Week 0 to Week 56** | | | |
| *n* | 54 | 48 | 40 |
| Absolute change (cm) | −11.444 (6.759) | −14.906 (7.946) | −3.094 (4.888) |
| **Change from Week 0 to Week 65** | | | |
| *n* | 29 | 26 | 19 |
| Absolute change (cm) | −11.643 (7.462) | −11.519 (8.937) | −3.829 (5.871) |
| **Change from Week 0 to Week 78** | | | |
| *n* | 54 | 49 | 39 |
| Absolute change (cm) | −10.496 (8.074) | −10.613 (9.290) | −4.015 (7.209) |

Data are mean (SD) unless otherwise specified. EAP, eligible analysis population; SD, standard deviation.

Supplementary Table S4 Percentage (%) and absolute (kg) change in body weight from Week 52 (EAP).

|  | **Tirzepatide 10 mg**  **(*n* = 57)** | **Tirzepatide 15 mg**  **(*n* = 51)** | **Placebo**  **(*n* = 44)** |
| --- | --- | --- | --- |
| **Change from Week 52 to Week 56** | | | |
| *n* | 53 | 46 | 38 |
| Percentage change (%) | 2.984 (2.752) | 2.626 (2.105) | 0.583 (2.043) |
| Absolute change (kg) | 2.192 (1.770) | 1.889 (1.535) | 0.547 (1.845) |
| Proportion of weight regain (%)* | 30.614 (31.983) | 18.253 (41.712) | 0.620 (164.837) |
| **Change from Week 52 to Week 65** | | | |
| *n* | 29 | 32 | 21 |
| Percentage change (%) | 4.013 (4.419) | 7.733 (6.319) | 0.273 (3.308) |
| Absolute change (kg) | 2.750 (3.197) | 5.367 (4.279) | 0.369 (3.227) |
| Proportion of weight regain (%) | 72.784 (174.469) | 56.156 (40.135) | 76.998 (79.137) |
| **Change from Week 52 to Week 78** | | | |
| *n* | 53 | 47 | 37 |
| Percentage change (%) | 9.061 (7.412) | 12.326 (9.881) | 1.825 (5.166) |
| Absolute change (kg) | 6.502 (5.604) | 8.723 (6.857) | 1.664 (5.085) |
| Proportion of weight regain (%) | 100.000 (0.000) | 100.000 (0.000) | 100.000 (0.000) |

Data are mean (SD) unless otherwise specified. *Proportion of weight regain = (body weight at Week 56/Week 65/Week 78 − body weight at Week 52)/(body weight at Week 78 − body weight at Week 52) × 100%. EAP, eligible analysis population; SD, standard deviation.

Supplementary Table S5 Absolute change in waist circumference (cm) from Week 52 (EAP).

|  | **Tirzepatide 10 mg (*n* = 57)** | **Tirzepatide 15 mg (*n* = 51)** | **Placebo (*n* = 44)** |
| --- | --- | --- | --- |
| **Change from Week 52 to Week 56** | | | |
| *n* | 53 | 46 | 38 |
| Absolute change (cm) | 1.250 (2.290) | 1.098 (1.649) | 0.243 (1.434) |
| **Change from Week 52 to Week 65** | | | |
| *n* | 29 | 26 | 19 |
| Absolute change (cm) | 1.478 (4.598) | 2.971 (5.750) | 0.974 (4.182) |
| **Change from Week 52 to Week 78** | | | |
| *n* | 53 | 47 | 37 |
| Absolute change (cm) | 2.891 (7.481) | 5.526 (6.523) | −0.516 (5.582) |

Data are mean (SD) unless otherwise specified. EAP, eligible analysis population; SD, standard deviation.

**Supplementary Figure S1** Patient flow diagram.


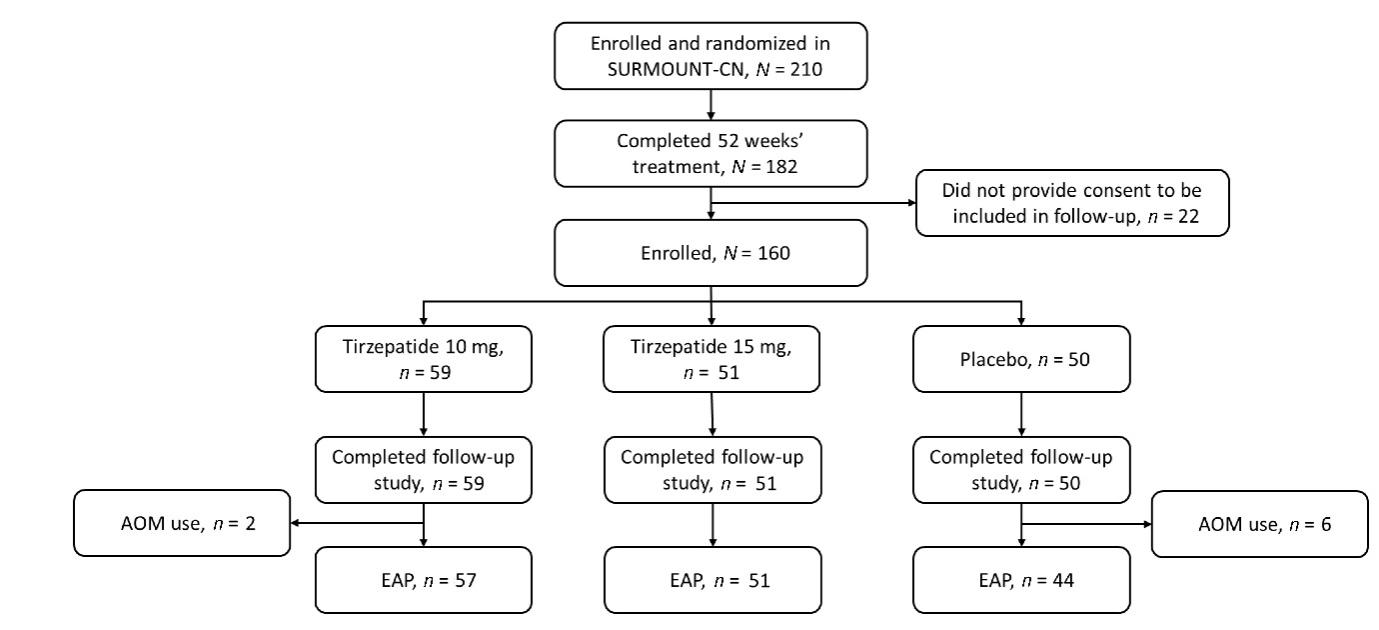


AOM, anti-obesity medication; EAP, eligible analysis population.
